# (*E*)-4-Hydr­oxy-*N*′-(2-hydr­oxy-4-methoxy­benzyl­idene)benzohydrazide monohydrate

**DOI:** 10.1107/S1600536808042888

**Published:** 2008-12-20

**Authors:** Nooraziah Mohd Lair, Hapipah Mohd Ali, Seik Weng Ng

**Affiliations:** a Department of Chemistry, University of Malaya, 50603 Kuala Lumpur, Malaysia

## Abstract

The Schiff base mol­ecule of the title compound, C_15_H_14_N_2_O_4_·H_2_O, adopts a *trans* configuration with respect to the C=N double bond; the Schiff base itself is almost planar (r.m.s. deviation for all non-H atoms = 0.040 Å). The amido N atom is the hydrogen-bond donor to the water mol­ecule, which is the hydrogen-bond donor to the hydr­oxy groups of two neighboring mol­ecules. One of the hydroxyl groups acts as an intra­molecular and the other as an inter­molecular hydrogen-bond donor.

## Related literature

For the structure of (*E*)-4-chloro-*N*′-(2-hydr­oxy-3-methoxy­benzyl­idene)benzo­hydrazide, which crystallizes as a monohydrate, see: Cui *et al.* (2007 ▶). For a series of similar compounds, see: Lu *et al.* (2008*a* ▶,*b* ▶,*c* ▶). For this and other compounds with anti­malarial properties, see: Melnyk *et al.* (2006 ▶).
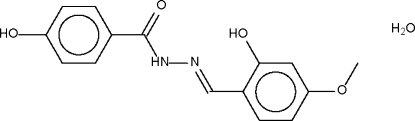


## Experimental

### 

#### Crystal data

C_15_H_14_N_2_O_4_·H_2_O
*M* *_r_* = 304.30
Monoclinic, 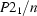

*a* = 7.1763 (2) Å
*b* = 16.6507 (5) Å
*c* = 12.1828 (4) Å
β = 98.022 (2)°
*V* = 1441.48 (8) Å^3^
*Z* = 4
Mo *K*α radiation
μ = 0.11 mm^−1^
*T* = 100 (2) K
0.16 × 0.04 × 0.04 mm

#### Data collection

Bruker SMART APEX diffractometer
Absorption correction: none
13327 measured reflections
3315 independent reflections
1903 reflections with *I* > 2σ(*I*)
*R*_int_ = 0.053

#### Refinement

*R*[*F* ^2^ > 2σ(*F* ^2^)] = 0.060
*wR*(*F* ^2^) = 0.180
*S* = 1.05
3315 reflections
200 parameters
H-atom parameters constrained
Δρ_max_ = 0.66 e Å^−3^
Δρ_min_ = −0.43 e Å^−3^

### 

Data collection: *APEX2* (Bruker, 2007 ▶); cell refinement: *SAINT* (Bruker, 2007 ▶); data reduction: *SAINT*; program(s) used to solve structure: *SHELXS97* (Sheldrick, 2008 ▶); program(s) used to refine structure: *SHELXL97* (Sheldrick, 2008 ▶); molecular graphics: *X-SEED* (Barbour, 2001 ▶); software used to prepare material for publication: *publCIF* (Westrip, 2009 ▶).

## Supplementary Material

Crystal structure: contains datablocks global, I. DOI: 10.1107/S1600536808042888/bt2836sup1.cif

Structure factors: contains datablocks I. DOI: 10.1107/S1600536808042888/bt2836Isup2.hkl

Additional supplementary materials:  crystallographic information; 3D view; checkCIF report

## Figures and Tables

**Table 1:** Hydrogen-bond geometry (Å, °)

*D*—H⋯*A*	*D*—H	H⋯*A*	*D*⋯*A*	*D*—H⋯*A*
O1—H1⋯O2^i^	0.84	1.86	2.621 (3)	150
O3—H3⋯N2	0.84	1.94	2.575 (3)	132
O5—H51⋯O1^ii^	0.84	2.03	2.833 (3)	160
O5—H52⋯O3^iii^	0.84	2.27	3.070 (4)	160
N1—H11⋯O5	0.88	2.05	2.883 (3)	158

Symmetry codes: (i) 
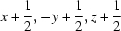
; (ii) 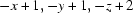; (iii) 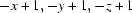.

**Fig. 1.**
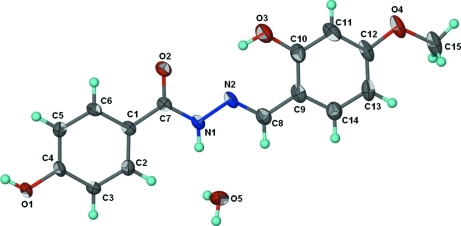
Anisotropic displacement ellipsoid plot (Barbour, 2001) of C15H14N2O4.H2O at the 70% probability level. Hydrogen atoms are drawn as spheres of arbitrary radius.

## supplementary crystallographic information

### Experimental

2-Hydroxy-3-methoxybenzaldehyde (0.30 g, 2 mmol) and 4-hydroxybenzohydrazide (0.30 g, 2 mmol) were heated in an ethanol-methanol mixture (50 ml) for 2 h. The solvent was removed and the resulting compound recrystallized from ethanol.

### Refinement

Carbon-bound H-atoms were placed in calculated positions (C—H 0.95–0.98 Å) and were included in the refinement in the riding model approximation, with *U*(H) set to 1.2–1.5*U*(C). The oxygen- and nitrogen-bound ones were located in a difference Fourier map, and were refined with distance restraints (O–H 0.84±0.01, N–H 0.88±0.01 Å); their isotropic displacement parameters were freely refined.

### Crystal data

**Table:**

C_15_H_14_N_2_O_4_·H_2_O	*F*(000) = 640
*M**_r_* = 304.30	*D*_x_ = 1.402 Mg m^−^^3^
Monoclinic, *P*2_1_/*n*	Mo *K*α radiation, λ = 0.71073 Å
Hall symbol: -P 2yn	Cell parameters from 1918 reflections
*a* = 7.1763 (2) Å	θ = 2.4–27.3°
*b* = 16.6507 (5) Å	µ = 0.11 mm^−^^1^
*c* = 12.1828 (4) Å	*T* = 100 K
β = 98.022 (2)°	Prism, yellow
*V* = 1441.48 (8) Å^3^	0.16 × 0.04 × 0.04 mm
*Z* = 4	

### Data collection

**Table:**

Bruker SMART APEX diffractometer	1903 reflections with *I* > 2σ(*I*)
Radiation source: fine-focus sealed tube	*R*_int_ = 0.053
graphite	θ_max_ = 27.5°, θ_min_ = 2.1°
ω scans	*h* = −9→9
13327 measured reflections	*k* = −21→21
3315 independent reflections	*l* = −15→15

### Refinement

**Table:**

Refinement on *F*^2^	Primary atom site location: structure-invariant direct methods
Least-squares matrix: full	Secondary atom site location: difference Fourier map
*R*[*F*^2^ > 2σ(*F*^2^)] = 0.060	Hydrogen site location: inferred from neighbouring sites
*wR*(*F*^2^) = 0.180	H-atom parameters constrained
*S* = 1.05	*w* = 1/[σ^2^(*F*_o_^2^) + (0.0687*P*)^2^ + 1.4764*P*] where *P* = (*F*_o_^2^ + 2*F*_c_^2^)/3
3315 reflections	(Δ/σ)_max_ = 0.001
200 parameters	Δρ_max_ = 0.66 e Å^−^^3^
0 restraints	Δρ_min_ = −0.43 e Å^−^^3^

### Fractional atomic coordinates and isotropic or equivalent isotropic displacement parameters (Å^2^)

**Table:**

	*x*	*y*	*z*	*U*_iso_*/*U*_eq_	
O1	0.7450 (3)	0.31702 (12)	1.09538 (15)	0.0271 (5)	
H1	0.7745	0.2698	1.1150	0.041*	
O2	0.3884 (3)	0.32742 (11)	0.58919 (15)	0.0246 (5)	
O3	0.1758 (3)	0.41831 (13)	0.32215 (18)	0.0444 (6)	
H3	0.2262	0.4067	0.3866	0.067*	
O4	−0.1014 (3)	0.59721 (14)	0.05179 (16)	0.0359 (6)	
O5	0.4249 (4)	0.61425 (14)	0.73043 (18)	0.0495 (7)	
H51	0.4027	0.6363	0.7892	0.074*	
H52	0.5420	0.6108	0.7317	0.074*	
N1	0.3620 (3)	0.45875 (14)	0.62786 (18)	0.0208 (5)	
H11	0.3800	0.4987	0.6754	0.025*	
N2	0.2800 (3)	0.47111 (14)	0.51985 (17)	0.0218 (5)	
C1	0.4997 (3)	0.36829 (15)	0.7739 (2)	0.0174 (5)	
C2	0.5351 (4)	0.42869 (16)	0.8536 (2)	0.0204 (6)	
H2	0.5012	0.4826	0.8345	0.024*	
C3	0.6192 (4)	0.41082 (16)	0.9602 (2)	0.0216 (6)	
H3A	0.6449	0.4524	1.0135	0.026*	
C4	0.6653 (3)	0.33229 (16)	0.9886 (2)	0.0197 (6)	
C5	0.6322 (4)	0.27144 (16)	0.9108 (2)	0.0214 (6)	
H5A	0.6655	0.2176	0.9305	0.026*	
C6	0.5504 (4)	0.28976 (16)	0.8043 (2)	0.0208 (6)	
H6	0.5284	0.2481	0.7508	0.025*	
C7	0.4134 (3)	0.38292 (16)	0.6579 (2)	0.0191 (6)	
C8	0.2221 (4)	0.54217 (18)	0.4913 (2)	0.0231 (6)	
H8	0.2362	0.5848	0.5436	0.028*	
C9	0.1350 (4)	0.55631 (18)	0.3780 (2)	0.0235 (6)	
C10	0.1117 (4)	0.49462 (18)	0.2982 (2)	0.0289 (7)	
C11	0.0295 (4)	0.5107 (2)	0.1905 (2)	0.0329 (7)	
H11A	0.0125	0.4689	0.1371	0.040*	
C12	−0.0278 (4)	0.5882 (2)	0.1614 (2)	0.0281 (7)	
C13	−0.0097 (4)	0.64983 (19)	0.2380 (2)	0.0290 (7)	
H13	−0.0519	0.7025	0.2177	0.035*	
C14	0.0714 (4)	0.63284 (18)	0.3450 (2)	0.0270 (6)	
H14	0.0843	0.6749	0.3982	0.032*	
C15	−0.1581 (4)	0.6759 (2)	0.0151 (3)	0.0385 (8)	
H15A	−0.2026	0.6749	−0.0647	0.058*	
H15B	−0.0508	0.7127	0.0299	0.058*	
H15C	−0.2598	0.6944	0.0549	0.058*	

### Atomic displacement parameters (Å^2^)

**Table:**

	*U*^11^	*U*^22^	*U*^33^	*U*^12^	*U*^13^	*U*^23^
O1	0.0379 (11)	0.0266 (11)	0.0146 (9)	0.0096 (9)	−0.0039 (8)	0.0015 (8)
O2	0.0309 (10)	0.0230 (10)	0.0188 (10)	−0.0048 (8)	−0.0008 (8)	−0.0005 (8)
O3	0.0701 (17)	0.0318 (13)	0.0264 (12)	−0.0012 (12)	−0.0107 (11)	0.0029 (10)
O4	0.0320 (12)	0.0538 (15)	0.0203 (10)	0.0041 (10)	−0.0018 (9)	0.0118 (10)
O5	0.0772 (18)	0.0431 (14)	0.0259 (12)	0.0265 (13)	−0.0009 (12)	−0.0119 (11)
N1	0.0228 (12)	0.0233 (12)	0.0152 (11)	0.0005 (9)	−0.0016 (9)	0.0003 (9)
N2	0.0209 (11)	0.0306 (13)	0.0132 (11)	−0.0014 (10)	0.0004 (9)	0.0039 (9)
C1	0.0136 (12)	0.0223 (13)	0.0164 (13)	−0.0033 (10)	0.0020 (10)	−0.0001 (11)
C2	0.0201 (13)	0.0213 (14)	0.0197 (13)	0.0023 (10)	0.0027 (10)	0.0031 (11)
C3	0.0260 (14)	0.0207 (14)	0.0172 (13)	0.0012 (11)	0.0001 (10)	−0.0035 (11)
C4	0.0192 (13)	0.0249 (14)	0.0144 (12)	0.0032 (11)	0.0007 (10)	0.0034 (11)
C5	0.0221 (13)	0.0201 (14)	0.0214 (13)	0.0022 (11)	0.0006 (11)	0.0011 (11)
C6	0.0217 (13)	0.0228 (14)	0.0170 (13)	−0.0005 (11)	−0.0004 (11)	−0.0033 (11)
C7	0.0173 (13)	0.0225 (14)	0.0176 (13)	−0.0027 (10)	0.0029 (10)	0.0000 (11)
C8	0.0197 (13)	0.0303 (15)	0.0195 (14)	0.0004 (11)	0.0035 (11)	0.0013 (12)
C9	0.0184 (13)	0.0327 (16)	0.0198 (14)	−0.0033 (11)	0.0043 (11)	0.0066 (12)
C10	0.0333 (16)	0.0286 (16)	0.0242 (14)	−0.0018 (13)	0.0013 (12)	0.0074 (13)
C11	0.0371 (17)	0.0391 (18)	0.0211 (15)	−0.0053 (14)	−0.0013 (12)	0.0008 (13)
C12	0.0192 (14)	0.0471 (19)	0.0177 (14)	−0.0008 (13)	0.0011 (11)	0.0108 (13)
C13	0.0237 (14)	0.0358 (17)	0.0269 (16)	0.0055 (12)	0.0018 (12)	0.0123 (13)
C14	0.0235 (14)	0.0327 (16)	0.0249 (15)	0.0039 (12)	0.0043 (12)	0.0033 (12)
C15	0.0314 (17)	0.057 (2)	0.0263 (16)	0.0062 (16)	0.0018 (13)	0.0173 (16)

### Geometric parameters (Å, °)

**Table:**

O1—C4	1.370 (3)	C3—H3A	0.9500
O1—H1	0.8400	C4—C5	1.385 (4)
O2—C7	1.243 (3)	C5—C6	1.382 (4)
O3—C10	1.369 (4)	C5—H5A	0.9500
O3—H3	0.8400	C6—H6	0.9500
O4—C12	1.374 (3)	C8—C9	1.452 (4)
O4—C15	1.426 (4)	C8—H8	0.9500
O5—H51	0.8400	C9—C14	1.394 (4)
O5—H52	0.8400	C9—C10	1.408 (4)
N1—C7	1.351 (3)	C10—C11	1.387 (4)
N1—N2	1.380 (3)	C11—C12	1.385 (4)
N1—H11	0.8800	C11—H11A	0.9500
N2—C8	1.286 (4)	C12—C13	1.381 (4)
C1—C6	1.393 (4)	C13—C14	1.381 (4)
C1—C2	1.396 (4)	C13—H13	0.9500
C1—C7	1.482 (3)	C14—H14	0.9500
C2—C3	1.386 (4)	C15—H15A	0.9800
C2—H2	0.9500	C15—H15B	0.9800
C3—C4	1.381 (4)	C15—H15C	0.9800
			
C4—O1—H1	119.9	N1—C7—C1	118.3 (2)
C10—O3—H3	120.0	N2—C8—C9	119.1 (3)
C12—O4—C15	117.3 (3)	N2—C8—H8	120.5
H51—O5—H52	108.8	C9—C8—H8	120.5
C7—N1—N2	117.5 (2)	C14—C9—C10	117.7 (3)
C7—N1—H11	121.3	C14—C9—C8	120.2 (3)
N2—N1—H11	121.3	C10—C9—C8	122.1 (3)
C8—N2—N1	118.3 (2)	O3—C10—C11	117.8 (3)
C6—C1—C2	118.4 (2)	O3—C10—C9	121.7 (3)
C6—C1—C7	117.8 (2)	C11—C10—C9	120.4 (3)
C2—C1—C7	123.8 (2)	C12—C11—C10	119.6 (3)
C3—C2—C1	120.7 (2)	C12—C11—H11A	120.2
C3—C2—H2	119.7	C10—C11—H11A	120.2
C1—C2—H2	119.7	O4—C12—C13	124.3 (3)
C4—C3—C2	119.8 (2)	O4—C12—C11	114.2 (3)
C4—C3—H3A	120.1	C13—C12—C11	121.5 (3)
C2—C3—H3A	120.1	C12—C13—C14	118.3 (3)
O1—C4—C3	117.9 (2)	C12—C13—H13	120.9
O1—C4—C5	121.5 (2)	C14—C13—H13	120.9
C3—C4—C5	120.5 (2)	C13—C14—C9	122.5 (3)
C6—C5—C4	119.4 (2)	C13—C14—H14	118.7
C6—C5—H5A	120.3	C9—C14—H14	118.7
C4—C5—H5A	120.3	O4—C15—H15A	109.5
C5—C6—C1	121.2 (2)	O4—C15—H15B	109.5
C5—C6—H6	119.4	H15A—C15—H15B	109.5
C1—C6—H6	119.4	O4—C15—H15C	109.5
O2—C7—N1	120.3 (2)	H15A—C15—H15C	109.5
O2—C7—C1	121.4 (2)	H15B—C15—H15C	109.5
			
C7—N1—N2—C8	176.8 (2)	N2—C8—C9—C14	−179.9 (3)
C6—C1—C2—C3	0.2 (4)	N2—C8—C9—C10	0.0 (4)
C7—C1—C2—C3	−178.9 (2)	C14—C9—C10—O3	177.7 (3)
C1—C2—C3—C4	−1.2 (4)	C8—C9—C10—O3	−2.2 (4)
C2—C3—C4—O1	−178.8 (2)	C14—C9—C10—C11	0.6 (4)
C2—C3—C4—C5	1.5 (4)	C8—C9—C10—C11	−179.3 (3)
O1—C4—C5—C6	179.6 (2)	O3—C10—C11—C12	−176.4 (3)
C3—C4—C5—C6	−0.7 (4)	C9—C10—C11—C12	0.8 (4)
C4—C5—C6—C1	−0.3 (4)	C15—O4—C12—C13	1.6 (4)
C2—C1—C6—C5	0.6 (4)	C15—O4—C12—C11	−178.2 (3)
C7—C1—C6—C5	179.7 (2)	C10—C11—C12—O4	178.0 (3)
N2—N1—C7—O2	0.7 (4)	C10—C11—C12—C13	−1.8 (4)
N2—N1—C7—C1	−179.3 (2)	O4—C12—C13—C14	−178.4 (3)
C6—C1—C7—O2	−0.7 (4)	C11—C12—C13—C14	1.4 (4)
C2—C1—C7—O2	178.4 (2)	C12—C13—C14—C9	0.0 (4)
C6—C1—C7—N1	179.3 (2)	C10—C9—C14—C13	−1.0 (4)
C2—C1—C7—N1	−1.6 (4)	C8—C9—C14—C13	178.9 (3)
N1—N2—C8—C9	−179.8 (2)		

### Hydrogen-bond geometry (Å, °)

**Table:**

*D*—H···*A*	*D*—H	H···*A*	*D*···*A*	*D*—H···*A*
O1—H1···O2^i^	0.84	1.86	2.621 (3)	150
O3—H3···N2	0.84	1.94	2.575 (3)	132
O5—H51···O1^ii^	0.84	2.03	2.833 (3)	160
O5—H52···O3^iii^	0.84	2.27	3.070 (4)	160
N1—H11···O5	0.88	2.05	2.883 (3)	158

Symmetry codes: (i) *x*+1/2, −*y*+1/2, *z*+1/2; (ii) −*x*+1, −*y*+1, −*z*+2; (iii) −*x*+1, −*y*+1, −*z*+1.
